# LRRK2 is a candidate prognostic biomarker for clear cell renal cell carcinoma

**DOI:** 10.1186/s12935-021-02047-y

**Published:** 2021-07-03

**Authors:** Chunxiu Yang, Jingjing Pang, Jian Xu, He Pan, Yueying Li, Huainian Zhang, Huan Liu, Shu-Yuan Xiao

**Affiliations:** 1 Department of Pathology, Zhongnan Hospital of Wuhan University, Wuhan, China; 2 Wuhan University Center for Pathology and Molecular Diagnostics, Wuhan, China; 3 Department of Pathology, University of Chicago Medicine, Chicago, IL USA

**Keywords:** LRRK2, Clear cell renal cell carcinoma, Bioinformatic analysis, Prognosis biomarker, HIF1A, EGFR, Tumorigenesis, Pathology

## Abstract

**Background:**

Clear cell renal cell carcinoma (ccRCC), derived from renal tubular epithelial cells, is the most common malignant tumor of the kidney. The study of key genes related to the pathogenesis of ccRCC has become important for gene target therapy.

**Methods:**

Bioinformatics analysis of The Cancer Genome Atlas (TCGA), the NCBI Gene Expression Omnibus (GEO) database, USUC Xena database, cBioPortal for Cancer Genomics, and MethSurv were performed to examine the aberrant genetic pattern and prognostic significance of leucine-rich repeat kinase 2 (LRRK2) expression and its relationship to clinical parameters. Immunohistochemistry and Western blot were performed to verify LRRK2 expression. The regulation of ccRCC tumor cell lines proliferation by LRRK2 was examined by CCK8 assay.

**Results:**

Bioinformatics analysis showed that LRRK2 expression was up-regulated and largely correlated with DNA methylation in ccRCC. The up-regulation of LRRK2 was confirmed in ccRCC tissue immunohistochemically and by protein analysis. The level of expression was related to gender, pathological grade, stage, and metastatic status of ccRCC patients. Meanwhile, Kaplan–Meier analysis showed that high expression of LRRK2 correlates to a better prognosis; knockdown of LRRK2 expression attenuated the proliferation ability of ccRCC tumor cell lines; protein–protein interaction network analysis showed that LRRK2 interacts with HIF1A and EGFR.

**Conclusion:**

We found that LRRK2 may play an important role in the tumorigenesis and progression of ccRCC. Our findings provided a potential predictor and therapeutic target in ccRCC.

**Supplementary Information:**

The online version contains supplementary material available at 10.1186/s12935-021-02047-y.

## Background

Renal cell carcinoma (RCC) represents a highly heterogeneous group of tumor, with clear cell renal cell carcinoma (ccRCC) being the most common histologic subtype [1]. Other subtypes include papillary RCC, chromophobe RCC, clear cell papillary RCC, and several other rare types. ccRCC is believed to derive from the proximal convoluted renal tubules. Its incidence increases with age, so it is more prevalent in the elderly, more common in male patients [2]. The common clinical manifestations of ccRCC are hematuria, pain, and renal mass. Histologically, ccRCC is characterized by high glycogen and lipid-rich cytoplasm. There is a genetic predisposition or hereditary factor associated with its tumorigenesis, with smoking, obesity, hypertension, chronic kidney disease, and other environmental factors being contributory [3, 4]. Although molecular genetic studies have shown that mutations of several genes are associated with the pathogenesis of ccRCC, including von Hippel-Lindau (VHL), set domain-containing 2 (SETD2), BRCA1-associated protein-1 (BAP1), polybromo-1 (PBRM1), and lysine-specific histone demethylase 5C (KDM5C) [5–8], additional genes are being identified to be related to RCC from cancer genomic studies, which may have prognostic, predictive and therapeutic relevance [9]. It has been shown that loss of the short arm of chromosome 3 is closely related to ccRCC, and the central molecular signature is the inactivation of the tumor suppressor VHL gene [5, 10]. Also, Myo-inositol monophosphatase 2 (IMPA2) downregulation is correlated with poor prognosis for ccRCC, and miR-25-mediated IMPA2 downregulation may be a potential therapeutic target for preventing the progression and metastatic of ccRCC [11].

Considering the high morbidity and mortality associated with ccRCC, it is essential to identify more molecular biomarkers that have diagnostic and prognostic value. In the present study, we aim to explore the expression of differential genes in renal cell carcinoma by analyzing data from independent public databases, and verifying the putative candidate by analyzing tumor and non-tumor control tissues. Our results show that leucine-rich repeat kinase 2 (LRRK2) is a prognostic biomarker for ccRCC.

## Materials and methods

### Data collection and bioinformatic analysis

All relevant data are available from the public domain databases. The RNA-seq FPKM data and corresponding clinicopathological data of 611 samples with renal clear cell carcinoma were obtained from The Cancer Genome Atlas databases (TCGA, https://portal.gdc.cancer.gov/). All of the 191 ccRCC sample series matrix files were downloaded from the NCBI Gene Expression Omnibus databases (GEO, https://www.ncbi.nlm.nih.gov/geo/), including 2 gene microarray datasets (GSE53757 and GSE71963). The background correction and normalization were performed using the Robust Multi-array Average (RMA) algorithm in R package “Affy”. Online genetic variation tool predictors USUC Xena (http://xena.ucsc.edu/) and cBioPortal for Cancer Genomics (http://www.cbioportal.org/) based on TCGA cohorts were used to analyze the RNA-seq data, somatic mutations, copy number alteration, and 450 K methylation array of LRRK2 in ccRCC. The survival analysis of DNA methylation of LRRK2 was carried out in MethSurv (https://biit.cs.ut.ee/methsurv/).

### Screening for hub genes

For the two GEO datasets, the R package “limma” was applied to identify differentially expressed genes (DEGs). The R package “EdgeR” was used to screen out DEGs based on the TCGA RNA-seq FPKM data. All DEGs were filtered by setting for *p* < 0.05 and |logFC| > 1 as cut-off criteria. The adjacency matrix was converted to topological overlap matrix (TOM) dissimilarity matrix (1-TOM) by “tomlikeity” arithmetic, and a scale-free topology of gene co-expression network was constructed through the R package “WGCNA” based on genes from the GSE71963 dataset. Correlation between the module eigengenes (MEs) and the clinical trait was calculated by Pearson’s correlation analysis to identify clinically significant modules. Subsequently, hub genes were considered those highly correlated with clinical traits as well as both the DEGs of GEO and TCGA datasets.

Prognostic analysis was performed by using the R package “survival” based on TCGA-KIRC clinicopathological data of 539 tumor and 72 normal samples. A univariate Cox regression analysis was used to evaluate the association between the hub gene expression and overall survival; prognostic-related genes were defined with a *p* < 0.001 cutoff. STRING (https://string-db.org/) was used to construct the protein–protein interaction (PPI) networks related to prognostically-related genes expressed with the retrieval condition of Organism: *Homo sapiens*, and a minimum required interaction score of medium confidence (0.400). Subsequently, a simple tabular text exported from STRING was input to the Cytoscape v3.7.1 to obtain the top10 nodes with the “betweenness” ranking method performed by cytoHubba, which were finally defined as key hub genes.

### Gene ontology (GO) terms and KEGG pathways

To further investigate the functional annotation and pathways of the prognostic-related genes, the R package “clusterProfiler” was adopted to carry out GO terms and KEGG pathway analyses, identified based on a threshold of adjusted *p* < 0.05.

### Validations through other online databases

An extensive search in PubMed about the key hub genes and ccRCC was conducted to exclude genes that had been previously reported. The remaining candidate genes were validated with the Human Protein Altas (HPA, https://www.proteinatlas.org/) by comparing the expression specificity and expression levels.

### Gene set enrichment analysis (GSEA)

Gene set enrichment analysis (GSEA) version 3.0 was used to predict the LRRK2 expression enriched pathways in ccRCC using the TCGA-KIRC datasets. ccRCC samples were divided into high and low expression level groups according to the median expression of LRRK2. The GeneChip matrix was analyzed after normalization and the number of random combinations was set to 1000. The functional annotation dataset c2.cp.kegg.v7.1.symbols.gmt was downloaded from the molecular signatures database (MsigDB, https://www.gsea-msigdb.org/gsea/msigdb) as a reference.

### Cell lines and culture

The human ccRCC cell line Caki-1 and human embryonic kidney cell line HEK-293T were purchased from the Cell Bank of the Chinese Academy of Sciences (Shanghai, China), the HK2, ACHN, A498 and 786-O cell lines were purchased from Procell Life Science & Technology Co., Ltd (Wuhan, China). These cell lines were authenticated via STR profiling and no mycoplasma contamination. The cell lines were incubated in McCoy’s 5A Medium (Gibco), Dulbecco’s modified Eagle’s medium (HyClone), RPMI 1640 medium (Hyclone) and minimal essential medium (MEM, Gibco), respectively, supplemented with 10% fetal bovine serum (Gibco) and 1% penicillin–streptomycin (Gibco) at 37 °C in 5% CO_2_ humidified atmosphere.

### Western blotting

Cells and tissues were lysed in lysis buffer supplemented with proteinase inhibitors. Protein samples were separated on SDS-PAGE and transferred to the PVDF membrane (Millipore, USA). The membranes were blocked with 5% non-fat milk for 1 h and incubated with the primary LRRK2 antibody (1:10,000, rabbit, ab133474, Abcam, USA) or GAPDH antibody (rabbit, 5174T, CST, USA) at 4 °C overnight, followed by washing and incubation with HRP-conjugated goat anti-rabbit antibody (1:40,000, ab205718, Abcam, USA) for 60 min at room temperature. The ECL western blotting detection kit (WBKLS0100, Millipore, USA) was used to detect the resultant bands. All experiments were performed in triplicate at least.

### RNA interference and cell growth assay

The lentiviral short hairpin RNA (shRNA) sequence targeting LRRK2 was purchased from Genechem Co., Ltd (Shanghai, China). Stable knockdown lines in A498 and 786-O cells were created by lentiviral particles containing shRNAs. 48 h post-infection, cells were passaged and selected with 2.0 μg/mL puromycin, and the stable lines were routinely used for all assays. Cell proliferation ability was measured by the cell count kit-8 (BS350A, Biosharp, China). Cells were seeded at a density of 5 × 10^3^ cells per well in a 96-well plate. After 24 h culture, 10 μL CCK8 solution was added to each well, and plate incubated for additional 4 h before measuring the absorbance at 450 nm wavelength using a microplate reader.

### Immunohistochemical staining

Formalin-fixed paraffin-embedded (FFPE) ccRCC and adjacent non-tumor tissues were obtained from our pathology archives, from September 2019 to August 2020 (detailed information in Additional file 1: Table S1). Sections in 5-μm-thickness were prepared from each tissue block for immunohistochemistry (IHC). Briefly, the paraffin sections were dewaxed and rehydrate for antigen retrieval and following elimination of the endogenous peroxidase activity, and then were blocked in 5% goat serum fluid for 1 h at room temperature. Next, the sections were incubated with primary LRRK2 antibody (1:500, rabbit, ab133474, Abcam, USA) at 4 °C overnight followed by visualization with the Dako EnVision DAB (Dako Diagnostics AG, Switzerland). For LRRK2 protein, staining localized in the cytoplasm is considered positive. Images were captured using an Olympus BX51 microscope equipped with a DP74 digital camera. Image-Pro Plus software (version 6.0) was used to assess the area and density of the stained regions, and the integrated optical density (IOD) value was obtained. The mean densitometry of the digital image (magnification, 20×) was designated as representative LRRK2 staining intensity. The signal density of the tissue areas from five randomly selected fields was counted in a blinded manner and subjected to statistical analysis.

### Statistical analysis

Bioinformatic statistics analyses were carried out by using R v 4.0.2. Wilcox test, Wilcoxon rank-sum test, and logistic regression analysis were used to examine the correlation between LRRK2 expression level and clinicopathological parameters of the TCGA ccRCC samples. Kaplan–Meier survival analysis and log-rank test were performed to draw the survival curve to evaluate the effect of LRRK2 on overall survival, with a 95% confidence interval and logarithmic rank *p*-value. Univariate and multivariate Cox regression analyses were applied to the comparison between LRRK2 expression and other clinicopathological parameters (age, gender, grade, and TNM stage) and to predict the independent prognostic-related hazard factors. Western blotting data were analyzed using the Image J software. Experimental statistics analyses were performed using Graphpad prism 8.

## Results

### Screening of differentially expressed genes in ccRCC

#### Identification of LRRK2 in ccRCC

**Fig. 1:**
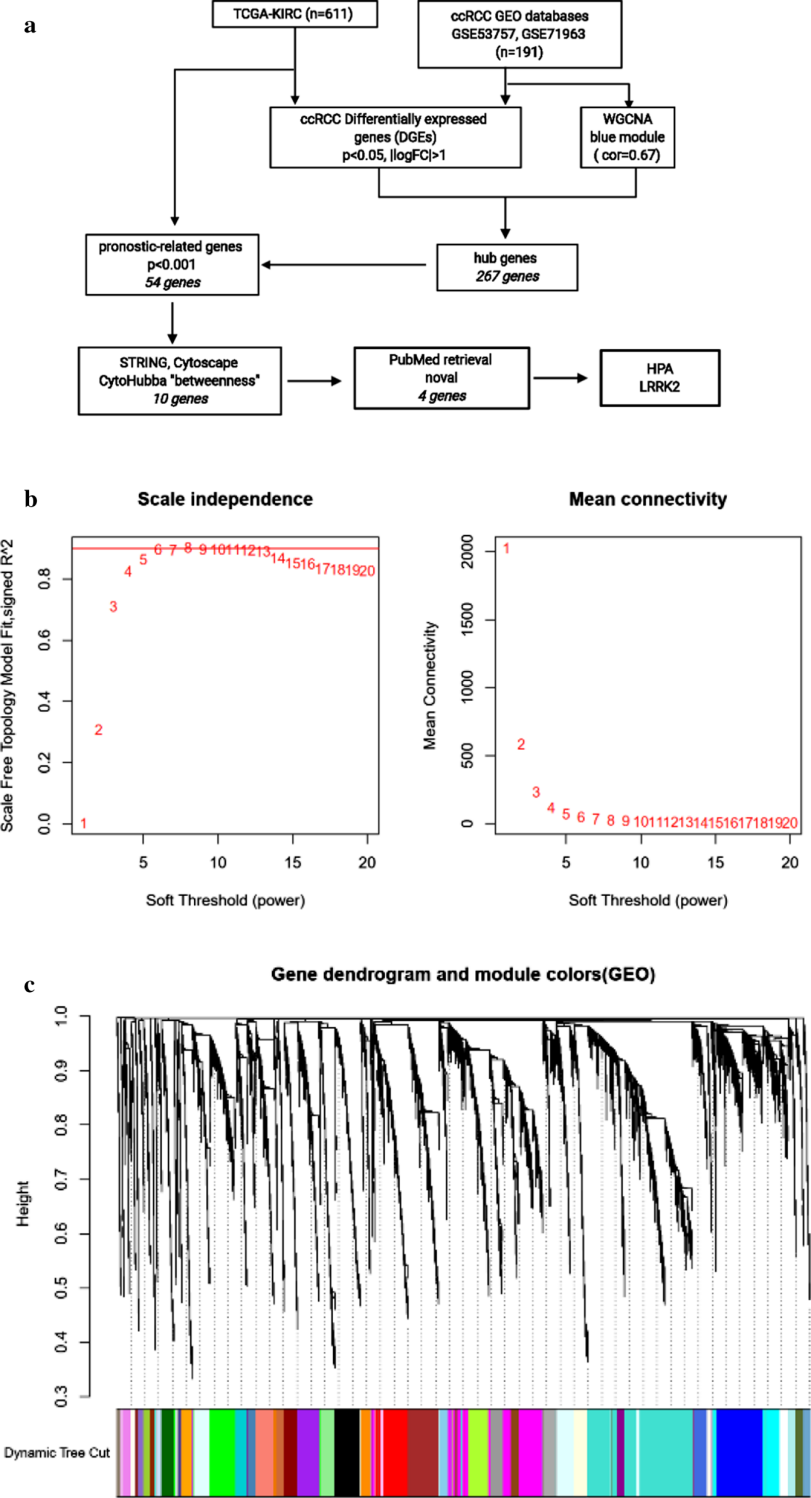

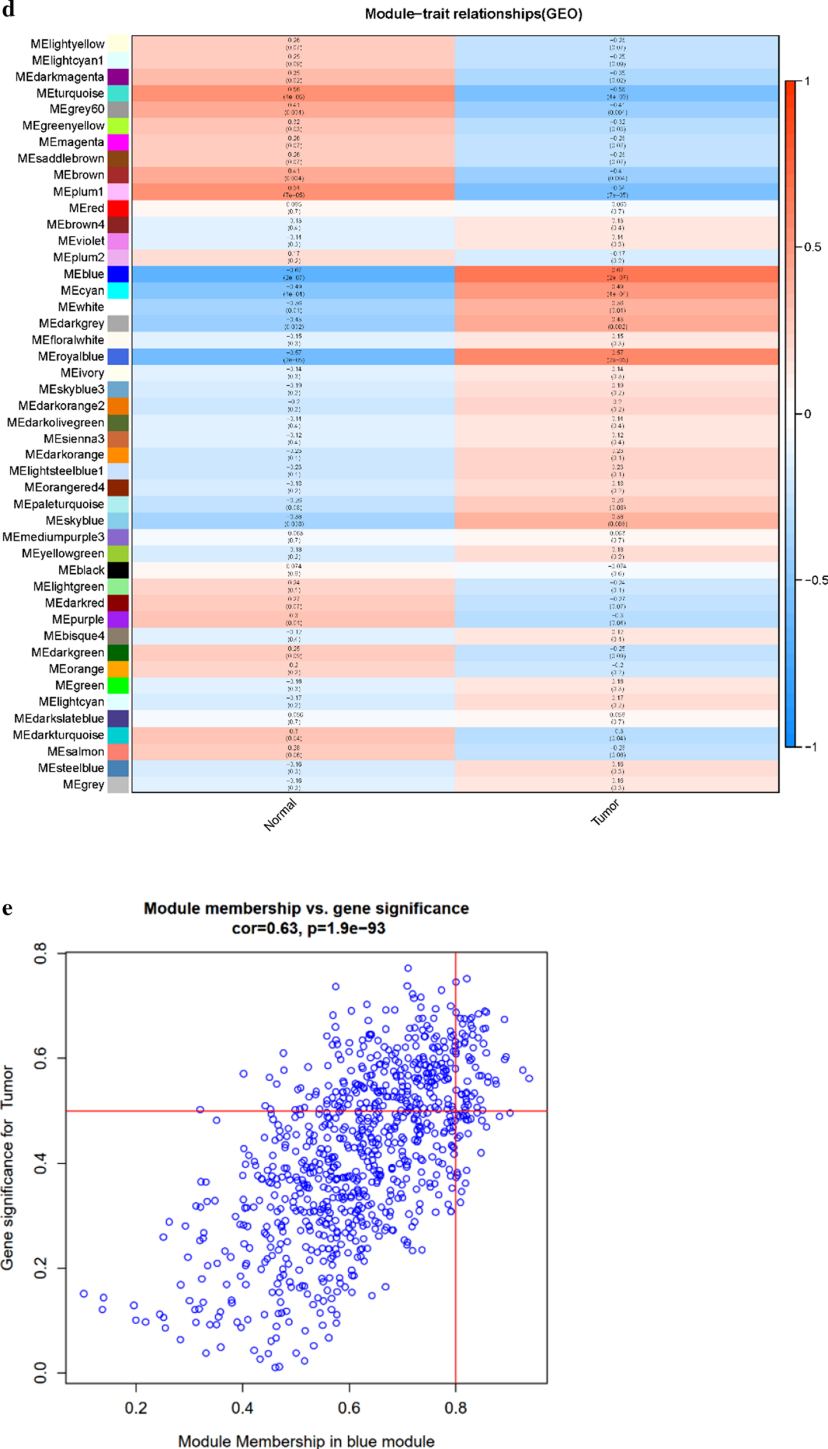
Modules of co-expressed genes in ccRCC. **a** Procedure for the selection and validation of the prognosis biomarkers in ccRCC; **b** identification of soft threshold power (β) for the scale-free network by using the “pickSoftThreshold” function; **c** cluster dendrogram of genes based on topological overlap matrix (TOM); **d** identification of modules associated with clinical traits; **e** scatter plot of module gene significance (GS)/module membership (MM) in the blue module

**Fig. 2:**
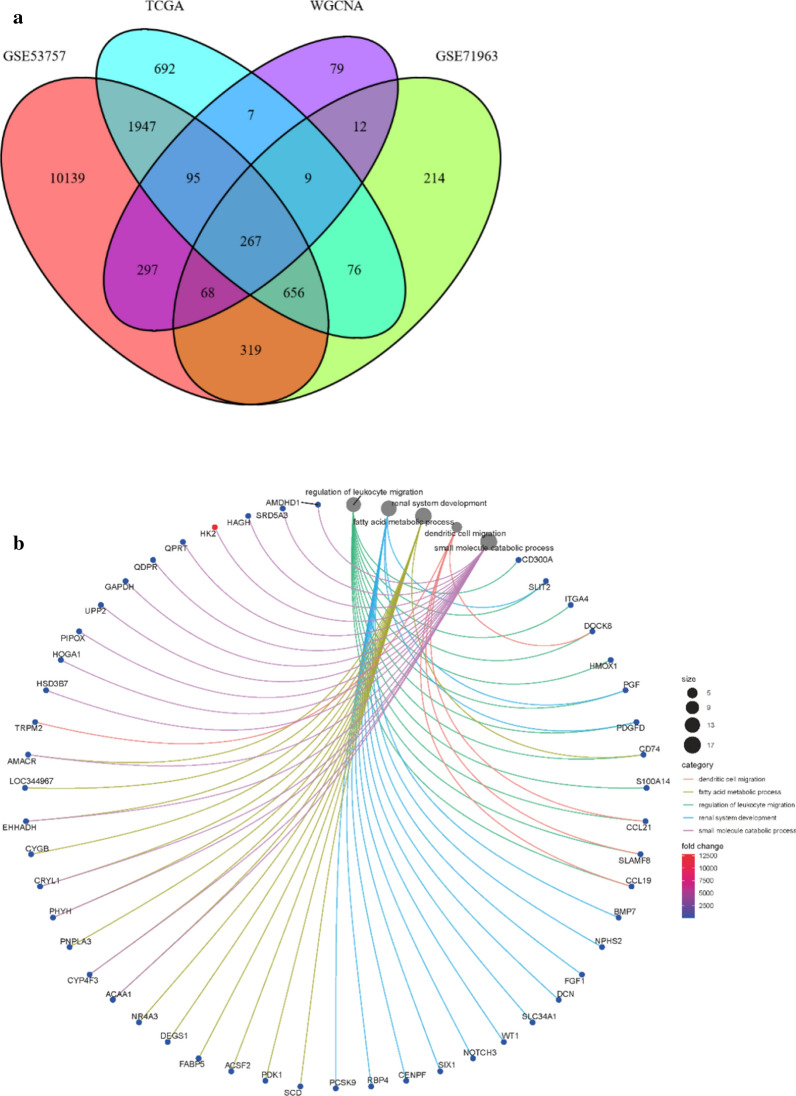

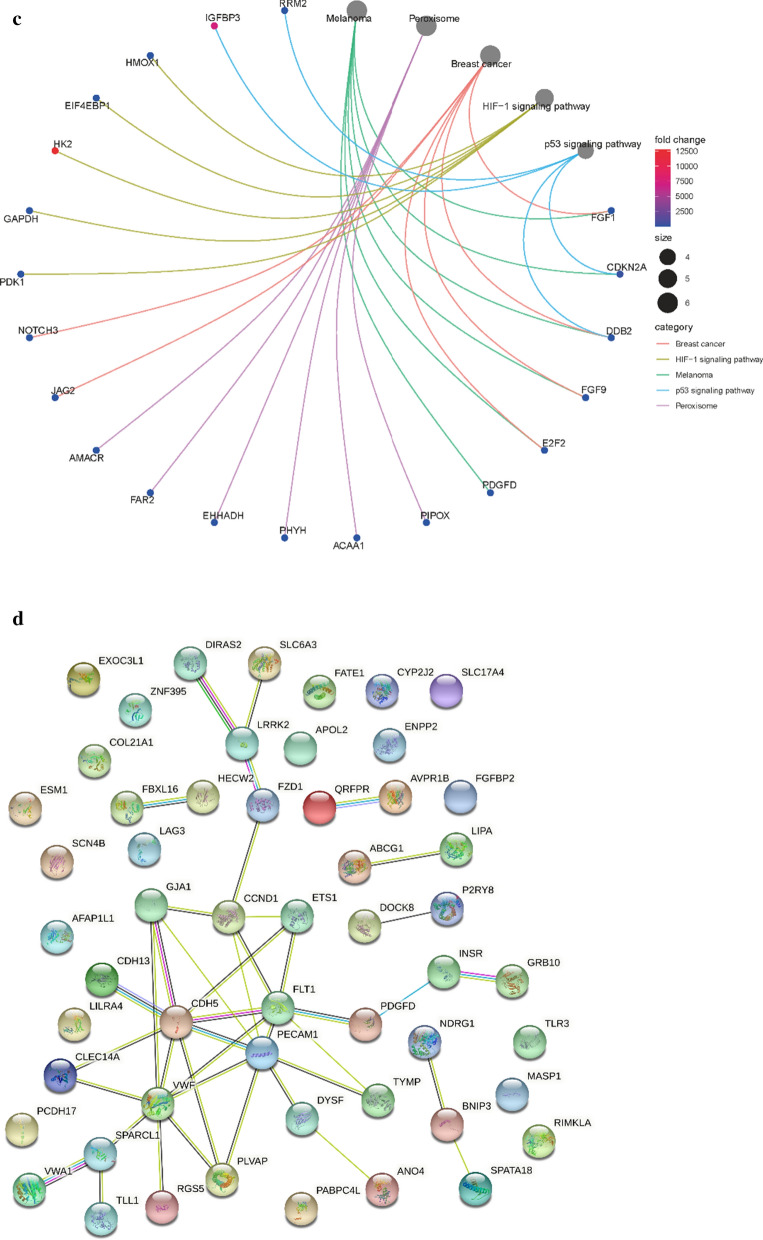

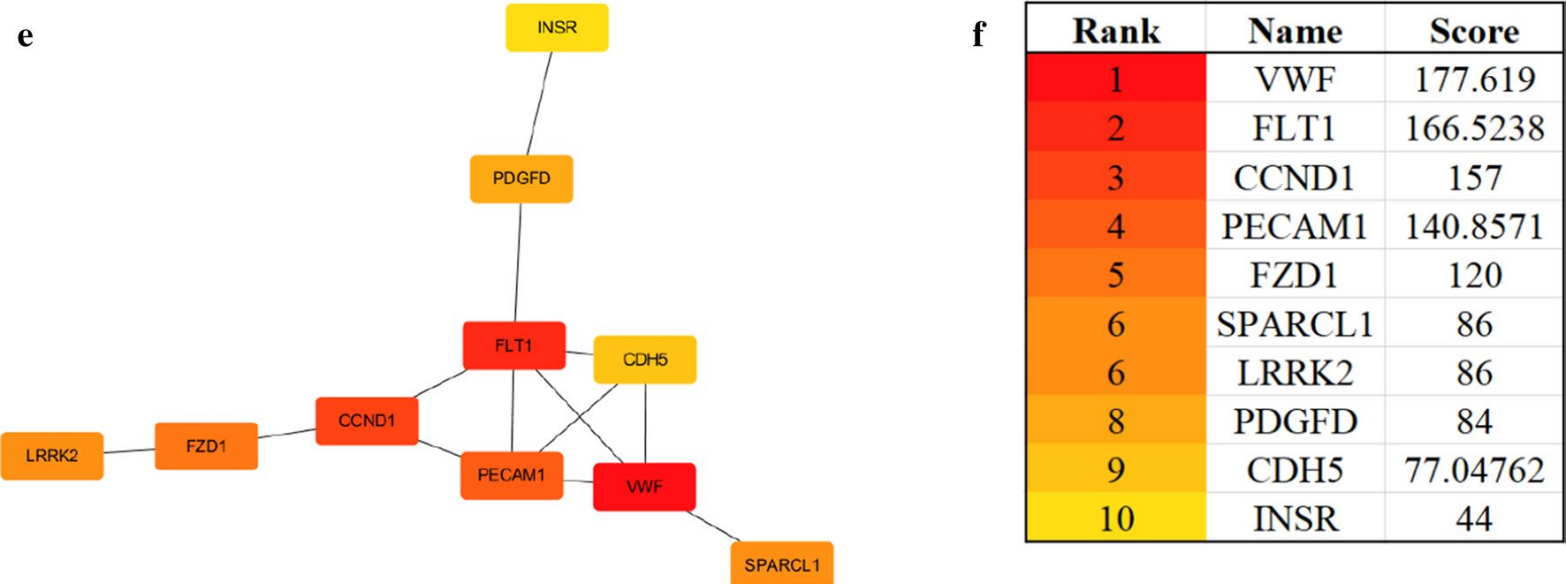
The screening procedures for key hub genes. **a** Venn diagram among TCGA DEGs, GEO DEGs, and genes from the blue module by WGCNA; **b**, **c** gene ontology terms and functional pathways of KEGG for identified intersected genes; **d** STRING analysis of prognostic-related genes; **e** top 10 nodes in STRING network ranked by “Betweenness” arithmetic; **f** the rank of 10 nodes. *TGCA* The Cancer Genome Atlas, *DEGs* differentially expressed genes, *GEO* Gene Expression Omnibus, *WGCNA* weighted gene co-expression network analysis, *KEGG* Kyoto Encyclopedia of Genes and Genomes, *STRING* search tool for recurring instances of neighboring genes

Bioinformatic approaches as described above were used to identify the target genes; the workflow of analysis is shown in Fig. 1a. First, DEGs of ccRCC were obtained from the two GEO datasets and TCGA-KIRC, with a total of 929 genes. Second, a soft threshold parameter β = 20 was selected (Fig. 1b) and a hierarchical clustering tree constructed by the correlation co-efficiency between genes from GSE71963, and different branches of clustering tree representing different gene modules in various colors (Fig. 1c). Based on the weighted correlation coefficient of genes, the genes were classified according to the expression patterns, and genes with similar patterns were classified as a module. In this way, we detected genes in the blue module exhibiting the most positive correlation with the tumor (Fig. 1d, e). Finally, with the intersection of the above- predicted genes, 267 hub genes were included in genes of both the blue module by WGCNA and DEGs in ccRCC (Fig. 2a). To further explore the pathway functional enrichment of the identified hub genes, we performed GO terms and KEGG pathways analyses. Based on GO annotations, functions of the hub genes were related to dendritic cell migration, fatty acid metabolic process, regulation of leukocyte migration, renal system development, and small molecule catabolic process. In addition, the top KEGG pathway enrichment included breast cancer, HIF-1 signaling pathway, melanoma, p53 signaling pathway, and peroxisomes (Fig. 2b, c). For the 267 hub genes, univariate regression analysis was performed with a *p*-value cut-off of 0.001, and 54 genes were filtered out as prognostic-related genes, which were mapped to the PPI network revealing 54 nodes and 42 edges (Fig. 2d). The top10 nodes were found based on the node degree calculated by cytoHubba in Cytoscape as the key hub genes (Fig. 2e, f).

Subsequently, PubMed searches for these 10 key hub genes and ccRCC allow us to exclude 8 genes, namely VWF, FLT1, CCND1, PECAM1, SPARCL1, FZD1, CDH5, and INSR, as already known to be associated with ccRCC. The remaining 2 genes, LRRK2 and PDGFD were considered as candidate novel prognosis-related differentially expressed genes for ccRCC. Validation of both LRRK2 and PDGFD were performed in the HPA database for expression specificity and expression levels. The LRRK2 expression is higher than PDGFD. Therefore, LRRK2 was chosen as a target gene for further study.

#### LRRK2 overexpression and genomic alteration in ccRCC

**Fig. 3:**
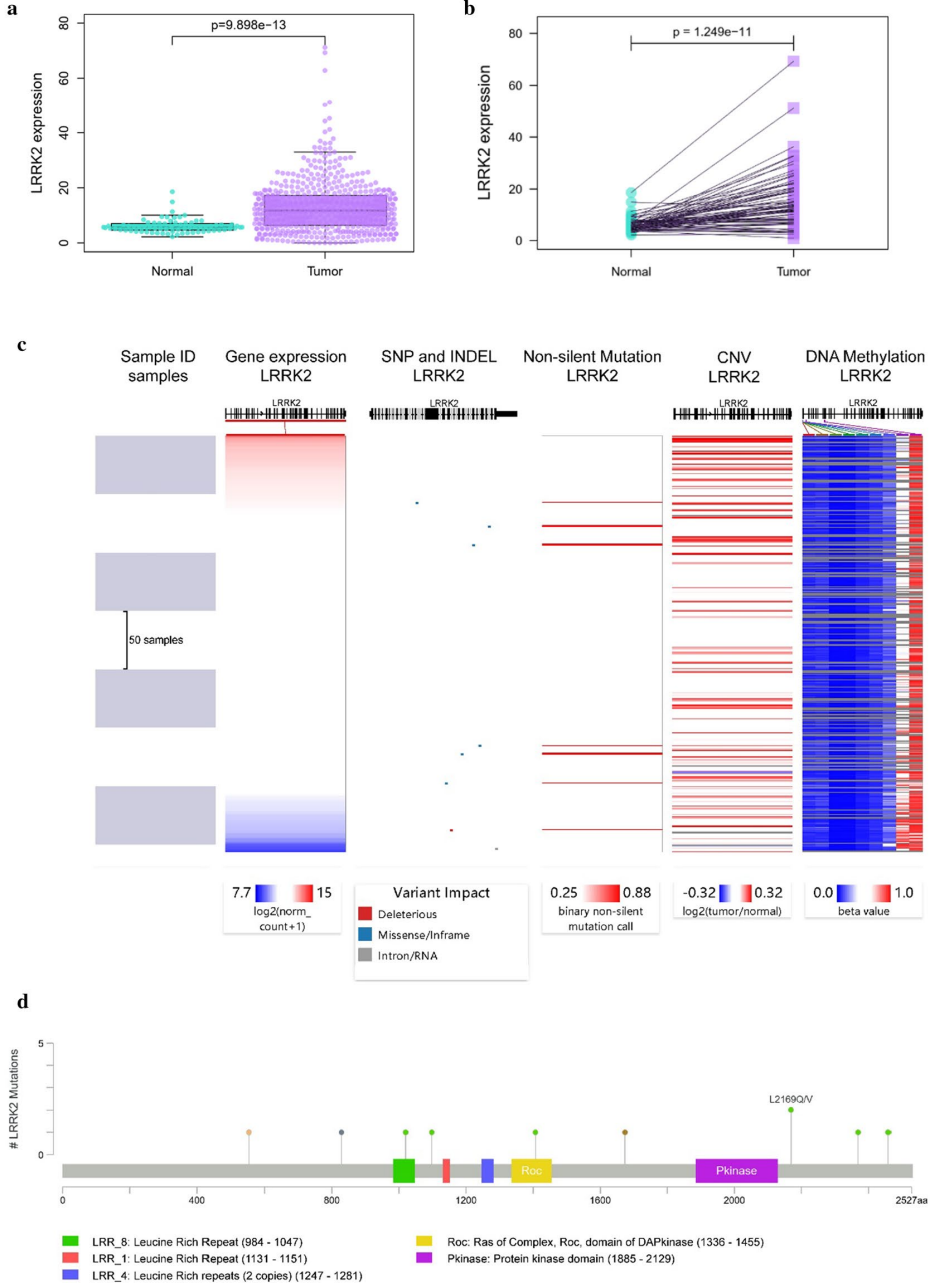

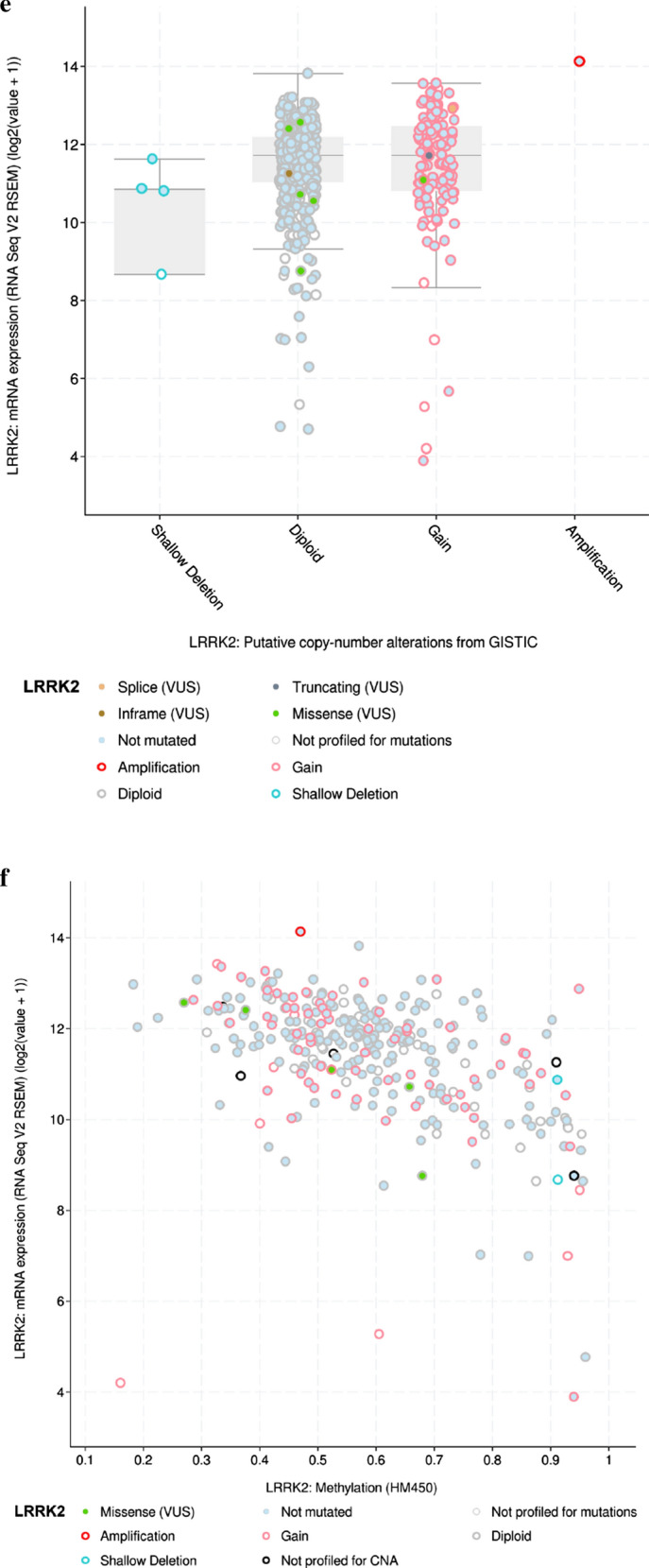

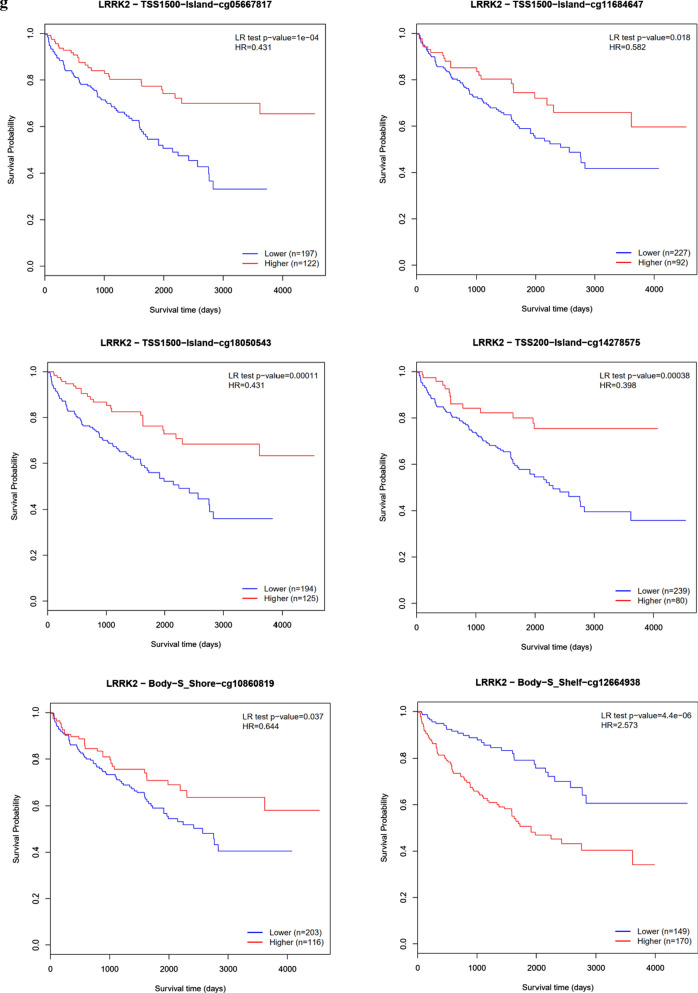
LRRK2 differential expression and genomic alteration in ccRCC tissues compared with normal control. **a** LRRK2 expression level in both tumor and normal tissues based on TCGA-KIRC datasets; **b** LRRK2 expression level in ccRCC tissues and paired adjacent non-tumor tissue. **c** The gene expression, somatic mutation, copy number variation (CNV), and DNA methylation from USUC Xena databases. **d** The mutation sites of LRRK2 in ccRCC patients. **e** The LRRK2 putative copy-number alterations according to cBioPortal for Cancer Genomics. **f** Negative correlations between the expression of LRRK2 and DNA methylation. **g** The survival curves of 6 methylated sites of LRRK2 in ccRCC patients. *ccRCC* clear cell renal cell carcinoma, *KIRC* kidney renal clear cell carcinoma

As described above, analyses from the TCGA RNA-seq data revealed LRRK2 to be significantly overexpressed in ccRCC as compared to that of non-tumor controls (p < 0.001). An example of the differentially expressed levels is shown in Fig. 3a, b. For further understanding the potential mechanisms of LRRK2 dysregulation, we examined for somatic mutations, including single nucleotide polymorphisms (SNPs), insertion–deletion (INDEL), copy number variation (CNV), and DNA methylation. Based on the genomic data of 260 ccRCC patients in the UCSC Xena database, we found that expression of LRRK2 is not significantly related to somatic mutations or CNV, but related to DNA methylation (Fig. 3c). With the cBioPortal, we conducted a more in-depth genomic analysis of 537 ccRCC patients for mutation sites of LRRK2 in tumor tissues (Fig. 3d) and found that LRRK2 tends to be slightly amplified (Fig. 3e), and confirmed that there is a negative correlation between LRRK2 expression and DNA methylation (Fig. 3f). According to the MethSurv, we found that the following six LRRK2 methylation sites are highly correlated with the survival of KIRC patients: cg05667817, cg11684647, cg18050543, cg14278575, cg10860819, and cg12664938. Therefore, DNA methylation is most likely involved in the aberrant expression of LRRK2 (Fig. 3g).

#### The correlation between LRRK2 and clinicopathological characteristics

**Fig. 4:**
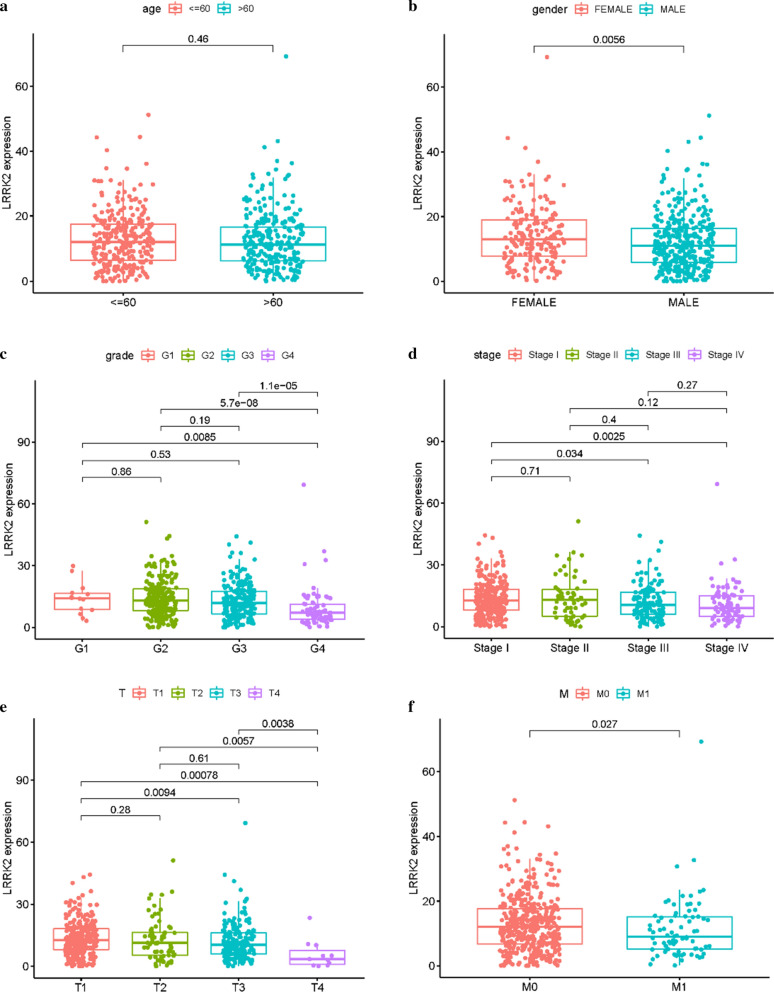
Relationship between LRRK2 expression and clinical characteristics. **a** Age (≤ 60 y and > 60 y); **b** gender; **c** grade; **d** stage; **e** tumor size and local growth (T); **f** occurrence of distant metastases (M)

A Wilcoxon signed-rank test and logistic regression analysis were used to analyze the correlation between LRRK2 expression and clinicopathological characteristics. The results suggest that the upregulation of LRRK2 expression is related to lower tumor grade, stage, and TMN, and LRRK2 overexpression is more frequently seen in women (Fig. 4). Logistic regression analysis showed that LRRK2 expression is associated with gender (male vs. female, OR = 0.67, 95% CI 0.47–0.96), grade (G1 vs. G4, OR = 0.16, 95% CI 0.04–0.53), stage (I vs. III, OR = 0.55, 95% CI 0.36–0.85; I vs. IV, OR = 0.44, 95% CI 0.26–0.72), T (T1 vs. T3, OR = 0.51, 95% CI 0.35–0.75; T1 vs. T4, OR = 0.07, 95% CI 0.00–0.39), M (M0 vs. M1, OR = 0.54, 95% CI 0.32–0.88) (Table 1).

**Table 1:** Logistic regression analysis of the relationship between LRRK2 expression and clinical characteristics

Clinical characteristics	Samples	OR (odd ratio)	*p-*value
Age (continuous)	537	0.99 (0.98–1.01)	0.214
Gender (female vs. male)	537	0.67 (0.47–0.96)	0.029*
Grade (G1 vs. G4)	520	0.16 (0.04–0.53)	0.004*
Stage (I vs. III)	534	0.55 (0.36–0.85)	0.007*
(I vs. IV)	534	0.44 (0.26–0.72)	0.001*
T (T1 vs. T3)	537	0.51 (0.35–0.75)	0.001*
(T1 vs. T4)	537	0.07 (0.00–0.39)	0.013*
M (M0 vs. M1)	505	0.54 (0.32–0.88)	0.015*

#### Prognostic value of LRRK2 based on TCGA-KIRC large cohorts

**Fig. 5:**
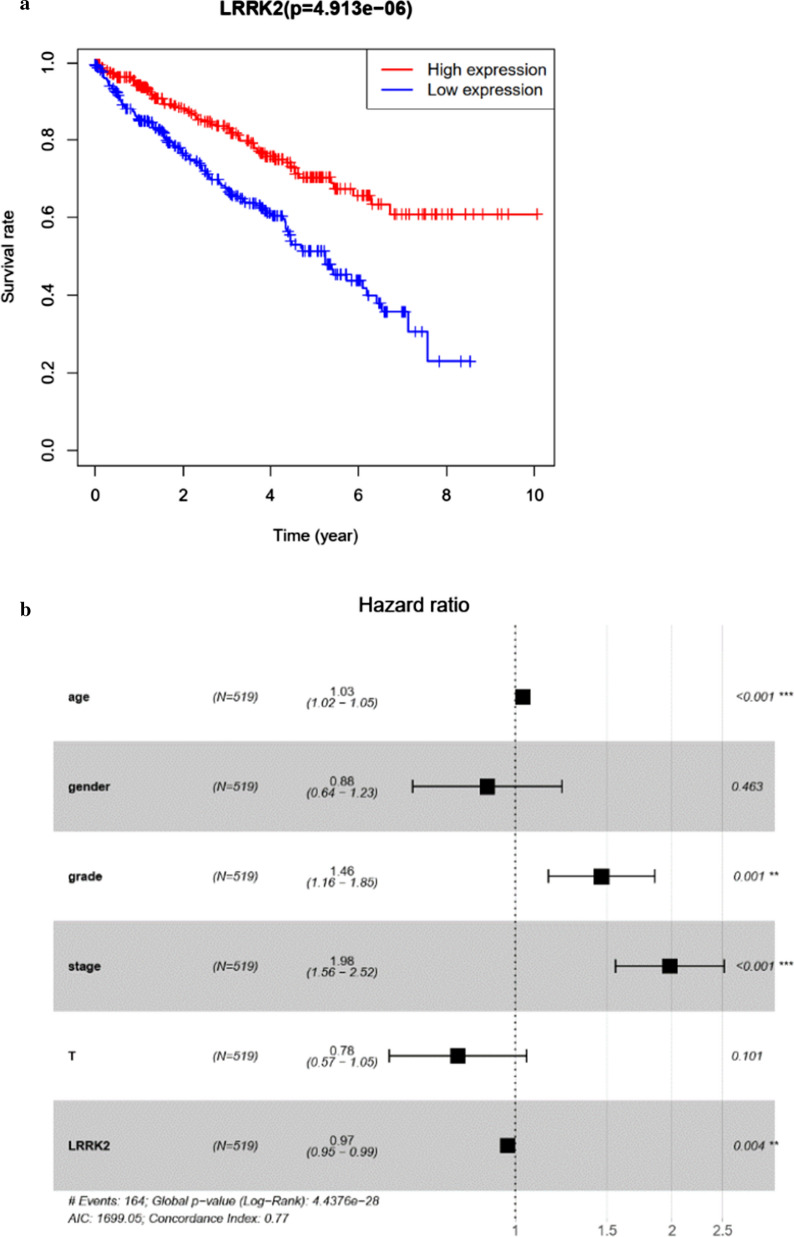
Prognostic analyses based on LRRK2 expression and overall survival. **a** Kaplan–Meier survival curve plotted based on a total of 530 patients; **b** multivariate Cox regression analysis of OS in TCGA cohorts grouped by the median of LRRK2 expression. *OS* overall survival

Kaplan–Meier survival curve from TCGA cohorts revealed that higher LRRK2 expression is associated with better prognosis in ccRCC patients (Fig. 5a). In multivariate regression analysis, the hazard ratio (HR) of LRRK2 expression (HR = 0.97, 95% CI 0.95–0.99) is less than 1, suggesting that higher LRRK2 expression correlates with better prognosis as improved overall survival (OS). In contrast, patient age (HR = 1.03, 95% CI 1.02–1.05), tumor grade (HR = 1.46, 95% CI 1.16–1.85) and stage (HR = 1.98, 95% CI 1.56–2.52) are correlated with poor prognosis, as can be expected (Fig. 5b, Table 2).

**Table 2:** Univariate and multivariate Cox regression analysis of overall survival in patients with ccRCC

Clinical parameter	Univariate Cox regression				Multivariate Cox regression			
	HR	95% CI		*p*	HR	95% CI		*p*
Age	1.03	1.02	1.05	0.000	1.03	1.02	1.05	0.000
Gender	0.95	0.69	1.31	0.773	0.88	0.64	1.23	0.463
Grade	2.32	1.88	2.86	0.000	1.46	1.16	1.85	0.001
Stage	1.90	1.67	2.18	0.000	1.98	1.56	2.52	0.000
T	1.95	1.65	2.31	0.000	0.78	0.57	1.05	0.101
LRRK2	0.95	0.93	0.97	0.000	0.97	0.95	0.99	0.004

*HR* hazard ratio, *CI* confidence interval

#### Gene set enrichment analysis (GSEA) for LRRK2

**Fig. 6:**
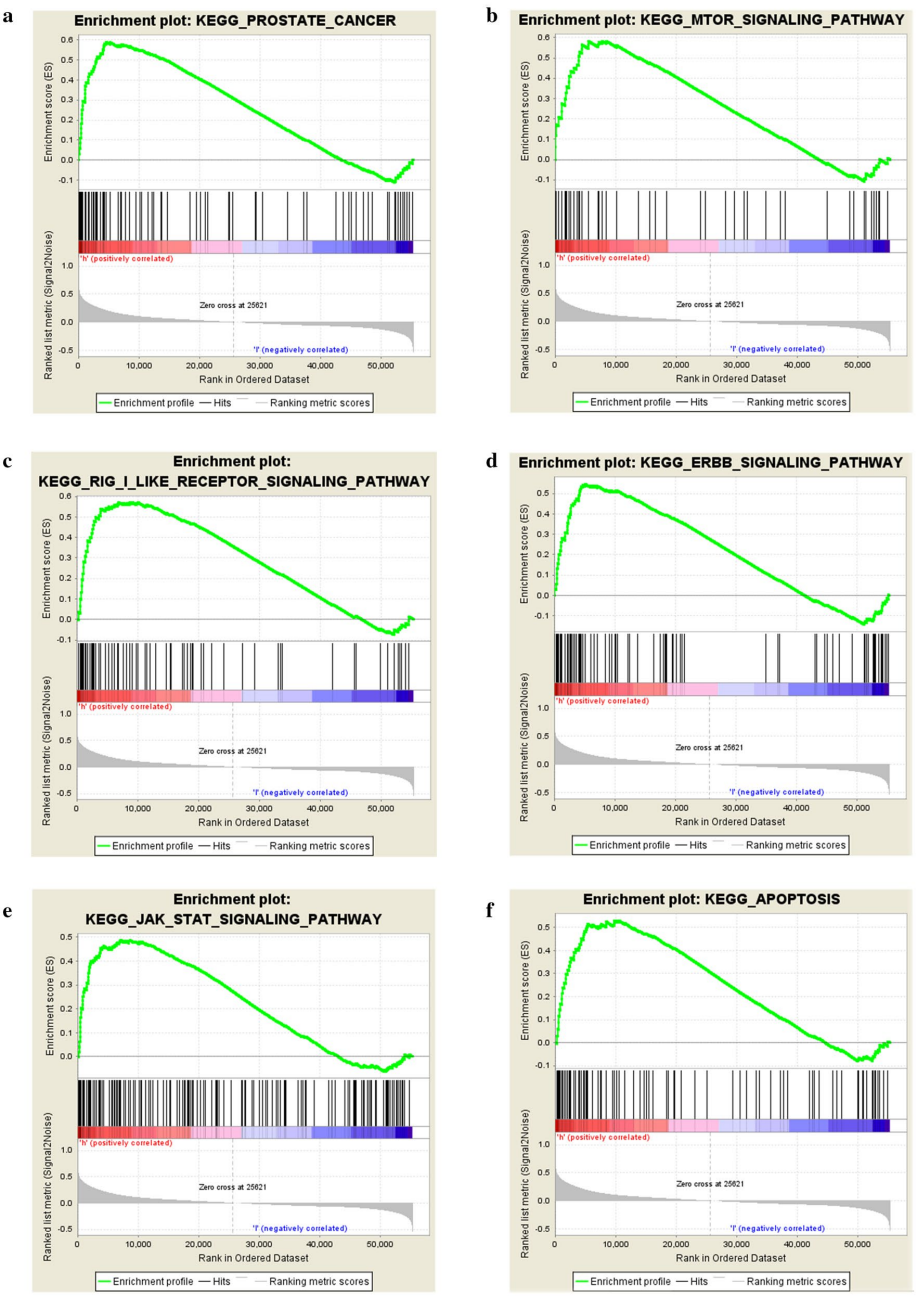
Six important enriched pathways involving LRRK2 in ccRCC according to GSEA. **a** Prostate cancer pathway; **b** mTOR signaling pathway; **c** RIG-I-like receptor signaling pathway; **d** ERBB signaling pathway; **e** JAK-STAT signaling pathway; **f** apoptosis pathway. *GSEA* Gene Set Enrichment Analysis

GSEA was conducted between two groups, the high LRRK2 expression and low LRRK2 expression, by calculating normalized enrichment score (NES) and selecting high LRRK2 expression enriched pathways (NOM *p*-val < 0.05, FDR *q*-val < 0.25). We identified “Prostate Cancer”, “MTOR signaling pathway”, “RIG-I-like receptor signaling pathway”, “ERBB signaling pathway”, “JAK-STAT signaling pathway” and “Apoptosis” as the potential functional enriched pathways modulated by LRRK2 (Table 3 and Fig. 6).

**Table 3:** Gene sets enriched in phenotype high

MSigDB collection	Gene set name	NES	NOM *p-val*	FDR *q-val*
c2.cp.kegg.v7.1.symbols.gmt	Prostate cancer	2.16	0.006	0.093
	MTOR signaling pathway	2.12	0.000	0.068
	RIG-I-like receptor signaling pathway	2.11	0.002	0.047
	ERBB signaling pathway	2.10	0.002	0.030
	JAK-STATE signaling pathway	2.06	0.002	0.031
	Apoptosis	1.99	0.011	0.032

*MSigDB* molecular signatures database, *NES* Normalized Enrichment Score, *FDR* false discovery rate

### The expression of LRRK2 is highly upregulated in patients with ccRCC tissue and cell lines

**Fig. 7:**
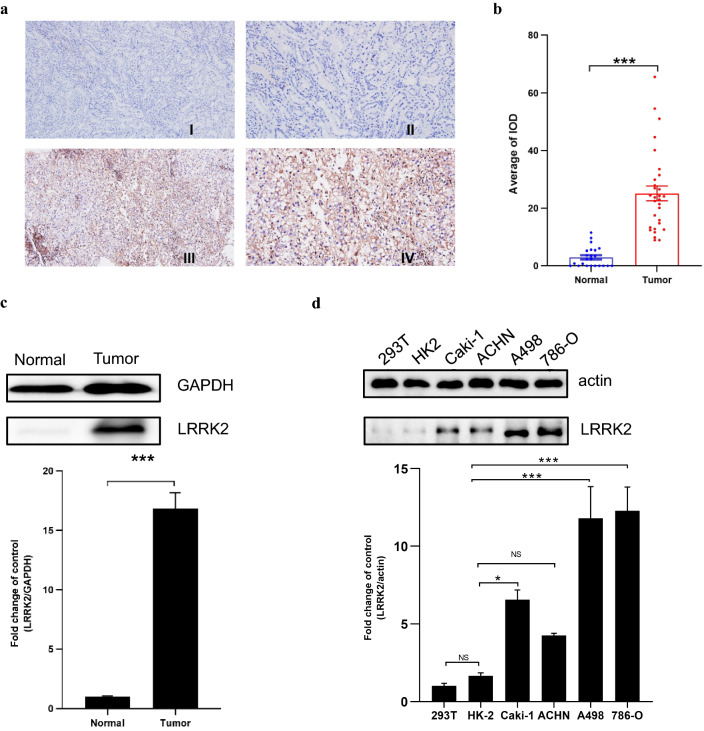
Expression in kidney tissue and cell lines. **a** Immunohistochemical (IHC) staining of LRRK2 in paired non-tumor tissue (n = 23, I. ×10 and II. ×20) and clear cell renal cell carcinoma tissues (n = 30, III. ×10 and IV. ×20); **b** IHC analysis of LRRK2 in non-tumor control and tumor tissues by image pro plus; **c** LRRK2 expression in ccRCC tissues and paired adjacent non-tumor tissue by western blotting (n = 3); **d** LRRK2 expression in HEK293T, HK-2 and ccRCC tumor cell lines by western blotting (n = 3)

To verify the findings from the above databases studies, we conducted a series of experimental validations. Overall, 30 cases of ccRCC were retrieved from the pathology archives, from 22 (73%) male and 8 (27%) female patients. The median age is 62 years (range 44–91 years). Immunohistochemically, as shown in Fig. 7a, LRRK2 is highly expressed in ccRCC, but hardly expressed in the adjacent non-tumor tissue. Based on the intensity and density of IHC staining, the LRRK2 IHC score of tumor tissue is significantly higher than that of the controls (Fig. 7b). Likewise, Western blotting results showed that the expression level of LRRK2 in ccRCC tissues was significantly higher than that in adjacent non-tumor tissues (Fig. 7c).

We further validated the expression of LRRK2 in the ccRCC cell lines by western blotting. Compared with the normal renal epithelial cell line HEK293T and HK2, the expression of LRRK2 is significantly upregulated in the ccRCC cell lines, including Caki-1, A498 and 786-O. The expression of LRRK2 in ACHN cell line showed similar trend, but the differences were not statistically significant (Fig. 7d).

### LRRK2 knockdown disrupts tumor cell proliferation

**Fig. 8:**
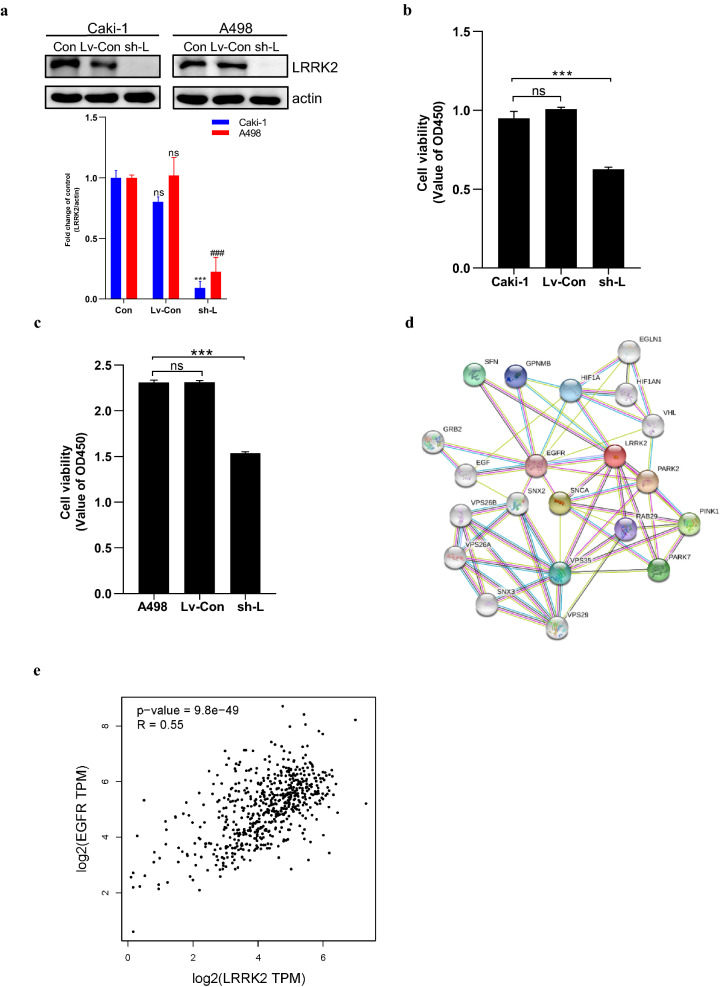
The function of LRRK2 in ccRCC tumor cell lines and protein–protein interaction of LRRK2. **a** Western blotting for LRRK2 expression in stable knockdown ccRCC tumor cell lines, Caki-1 and A498 cells were stably infected with lentiviral shRNA targeting LRRK2 (sh-L) or with a nontargeting control shRNA (Lv-Con). Values are expressed as mean ± SEM (n = 3). Within each group, values significantly different from control (Con) (p < 0.05) are indicated with an asterisk or pound key; **b** the proliferation ability of stable knockdown Caki-1 was uncovered by CCK8 assay; **c** the proliferation ability of stable knockdown A498 was uncovered by CCK8 assay; **d** the protein–protein interaction network of LRRK2 by STRING database; **e** correlation between LRRK2 and EGFR in ccRCC using the Gene Expression Profiling Interactive Analysis (GEPIA) database

We used a loss-of-function strategy in culture A498 and 786-O cells, using lentiviral RNA to determine the functional role of LRRK2 in ccRCC. The shRNA diminished LRRK2 protein levels in ccRCC tumor cell lines (Fig. 8a). Analysis of cell growth after stable knockdown LRRK2 demonstrates a restraint in cell growth compared with a control shRNA (Fig. 8b, c).

### Protein–protein interaction (PPI) network of LRRK2

Previous studies have shown that in addition to the loss of chromosome arm 3p, there can be loss of chromosome 14q in ccRCC. The latter contains *HIF1A* [12]. The PPI network prediction analysis using the STRING database showed that there is a potential interaction between LRRK2 and HIF1A (score = 0.941), indicating that LRRK2 may play a vital role in the pathogenesis of ccRCC (Fig. 8d). In addition, the PPI network prediction analysis showed a potential interaction between LRRK2 and EGFR (score = 0.921), and the interaction of LRRK2 and EGFR was analyzed using the Gene Expression Profiling Interactive Analysis (GEPIA) database, suggesting the positive correlation between them (Fig. 8e).

## Discussion

The prognosis of renal cell carcinoma (RCC) is difficult to predict. Therefore, it is essential to identify more reliable biomarkers to guide clinical management. In this study, we found that LRRK2 is upregulated in ccRCC, and high LRRK2 expression is associated with patient outcomes.

LRRK2 is a kinase encoded by the LRRK2 gene. It has a complex structure with multiple domains: a Ras of complex GTPase domain (ROC), a C-terminal of ROC domain (COR), and a Ser/Thr kinase domain [13]. The HPA database shows that LRRK2 protein is mainly expressed in renal tubular epithelial cells and immune cells. The biological functions of LRRK2 include protein translation, regulation of autophagy, and axonal degeneration induced by α-synuclein [13, 14]. By analyzing multiple databases for the expression of individual proteins, and confirming in clinical specimens as well as cell lines, we have found that LRRK2 is over-expressed in ccRCC. We speculate that LRRK2 can be a novel prognostic biomarker of renal cell carcinoma. Analysis of the TCGA datasets has shown that high expression of LRRK2 is related to the gender of the patients, tumor grade, stage, metastatic status, and prognosis of ccRCC patients.

Previously, research on LRRK2 were mainly focused on its role in Parkinson’s disease. A study showed that LRRK2 is amplified and overexpressed in papillary renal carcinoma, and down-regulation of LRRK2 in cultured tumor cells compromises cellular mesenchymal–epithelial transition factor (c-MET) signaling activation, which affects the growth and survival of tumor cells [15], but the expression of LRRK2 in clear cell renal cell carcinoma had not been studied. Our data showed that knockdown of LRRK2 expression attenuats the proliferative ability of ccRCC tumor cell lines, indicating that LRRK2 has a critical role in ccRCC tumor cell growth and proliferation, but the specific mechanisms needs further investigation. The protein–protein interaction network (PPI) analysis of the STRING database has shown that LRRK2 interacts with EGFR and HIF1A. The epidermal growth factor receptor (EGFR) signaling pathway plays a critical role in the pathogenesis and progression of renal cell carcinoma [16, 17], suggested that LRRK2 may play an important role in ccRCC as well. Moreover, HIF1A is a transcription factor that regulates the expression of several hypoxia-responsive genes, including vascular endothelial growth factor (VEGF) [18], platelet-derived growth factor (PDGF) [19], and glucose transporters GLUT1 and GLUT4 [20]. Hypoxia is a key step in the occurrence and development of renal cell carcinoma, which is mainly regulated by the tumor suppressor gene VHL, and the VHL-HIF1A-VEGFA protein axis is involved in the occurrence and development of renal cell carcinoma [21]. VHL mutation and inactivation can lead to the accumulation of HIF1A transcription factors, which can trigger VEGFA transcription to promote angiogenesis and play a key role in tumorigenesis and development [21, 22]. HIF1A is detected in about 70% of ccRCC and is closely related to patient survival [23]. In addition, HIF1A is necessary for the clear cell phenotype [22, 24]. Overall, we suspect that LRRK2 may play a role in the occurrence of, and influence the development of RCC by regulating HIF1A, and ultimately affect the survival of ccRCC patients. However, the exact mechanisms need to be further studied.

In conclusion, our results show that LRRK2 is expressed and up-regulated in ccRCC as identified by bioinformatics analysis and confirmed in tissue specimens, suggesting that LRRK2 may be a potential target for ccRCC treatment. However, additional studies are necessary for further elucidation of the mechanisms.

## Supplementary Information

**Additional file 1: Table S1.** Detailed information of ccRCC patients is listed.

## Data Availability

The datasets generated during the current study are available in the Gene Expression Omnibus (GEO) database (https://www.ncbi.nlm.nih.gov/geo/), the cancer genome atlas (TCGA) database (https://portal.gdc.cancer.gov/), Gene Expression Profiling Interactive Analysis (GEPIA) database (http://gepia2.cancer-pku.cn/#index), and STRING database (https://string-db.org/).

## Abbreviations

ccRCC: Clear cell renal cell carcinoma
TCGA: The Cancer Genome Atlas
GEO: Gene Expression Omnibus
LRRK2: Leucine-rich repeat kinase 2
GEPIA: Gene Expression Profiling Interactive Analysis
VHL: Von Hippel-Lindau
SETD2: Set domain-containing 2
BAP1: BRCA1-associated protein-1
PBRM1: Polybromo-1
KDM5C: Lysine-specific histone demethylase 5C
IMPA2: Myo-inositol monophosphatase 2
RMA: Robust Multi-array Average
DEGs: Differentially expressed genes
TOM: Topological overlap matrix
WGCNA: Weighted gene co-expression network analysis
KIRC: Kidney renal clear cell carcinoma
MEs: Module eigengenes
STRING: Search tool for recurring instances of neighboring genes
PPI: Protein–protein interaction
GO: Gene Ontology
KEGG: Kyoto Encyclopedia of Genes and Genomes
HPA: Human Protein Altas
GSEA: Gene set enrichment analysis
MsigDB: Molecular signatures database
FFPE: Formalin-fixed paraffin-embedded
IHC: Immunohistochemistry
IOD: Integrated optical density
SNPs: Single nucleotide polymorphisms
INDEL: Insertion–deletion
CNV: Copy number variation
HR: Hazard ratio
OS: Overall survival
RCC: Renal cell carcinoma
ROC: RAS of complex GTPase domain
COR: C-terminal of ROC domain
c-MET: Cellular mesenchymal–epithelial transition factor
EGFR: Epidermal growth factor receptor
VEGF: Vascular endothelial growth factor
PDGF: Platelet derived growth factor
