# Supplementary material for: LRRK2 is a candidate prognostic biomarker for clear cell renal cell carcinoma
Source: Cancer Cell Int. 2021 Jul 3;21:343. doi: 10.1186/s12935-021-02047-y (PMC8254929; doi:10.1186/s12935-021-02047-y)
Supplement: Supplementary file 1 — Additional file 1: Table S1. Detailed information of ccRCC patients is listed. [file 12935_2021_2047_MOESM1_ESM.docx]

Table S1. Detailed information of ccRCC patients is listed

| **Number** | **Gender** | **Age at surgery** | **TNM** | **Fuhrman Grade** |
| --- | --- | --- | --- | --- |
| 1 | Male | 61 | T1aNxMx | II |
| 2 | Male | 62 | T2aNxMx | III |
| 3 | Male | 65 | T3aNxMx | II |
| 4 | Male | 58 | T1aNxMx | II |
| 5 | Male | 62 | T3aN0Mx | III |
| 6 | Male | 75 | T4N1Mx | III, focal IV |
| 7 | Female | 67 | T1aNxMx | I |
| 8 | Male | 49 | T2aNxMx | III |
| 9 | Female | 67 | T1bNxMx | II |
| 10 | Female | 60 | T1NxMx | II |
| 11 | Male | 69 | T3aN0Mx | II |
| 12 | Male | 47 | T1bNxMx | IV |
| 13 | Male | 44 | T3aN0Mx | II |
| 14 | Male | 59 | T3aN0Mx | III |
| 15 | Male | 71 | T3aNxMx | II, focal III-IV |
| 16 | Male | 55 | T1bNxMx | III |
| 17 | Male | 64 | T3aN0Mx | III |
| 18 | Female | 67 | T1aNxMx | II |
| 19 | Male | 72 | T1bNxMx | II |
| 20 | Male | 65 | T3aN1Mx | III-IV |
| 21 | Female | 56 | T3aNxMx | II |
| 22 | Male | 91 | T1bNxMx | II |
| 23 | Male | 52 | T1bNxMx | I |
| 24 | Female | 56 | T1aNxMx | II |
| 25 | Male | 50 | T1aNxMx | II |
| 26 | Female | 51 | T1bNxMx | I |
| 27 | Male | 69 | Not given | II |
| 28 | Male | 67 | T1aNxMx | I |
| 29 | Male | 57 | Not given | II |
| 30 | Female | 72 | Not given | II |
